# Examining factors associated with catheter-defined unplanned dialysis initiation: a UK Renal Registry analysis

**DOI:** 10.1093/ckj/sfag287

**Published:** 2026-08-31

**Authors:** Winnie Magadi, Kate Birnie, Yoav Ben-Shlomo, Fergus Caskey, Shalini Santhakumaran

**Affiliations:** UK Renal Registry, UK Kidney Association, Bristol, UK; Population Health Sciences, Bristol Medical School, Bristol, UK; Population Health Sciences, Bristol Medical School, Bristol, UK; Population Health Sciences, Bristol Medical School, Bristol, UK; Population Health Sciences, Bristol Medical School, Bristol, UK; UK Renal Registry, UK Kidney Association, Bristol, UK

**Keywords:** CKD, dialysis, epidemiology, ethnicity, risk factors

## Abstract

**Background:**

In the UK, nearly half of patients with end stage kidney disease (ESKD) who initiate dialysis do so in an unplanned manner. A reduced quality of life, increased number of hospitalisations, and higher rates of mortality are just a few of the consequences of unplanned dialysis initiation (UDI). Our study examined factors associated with UDI in patients with ESKD in England, focusing on the effects of ethnicity and late presentation (LP).

**Patients and methods:**

We conducted a retrospective cohort study using data from the UK Renal Registry on 26 512 adult patients initiating dialysis between 2019 and 2023. UDI was defined as starting dialysis via a tunnelled or non-tunnelled line. We ran univariable and multivariable (multilevel) logistic regression analyses to examine risk factors of UDI, then tested for interactions between ethnicity and other key risk factors.

**Results:**

Just under half of the population had a UDI (49%), with Black and ‘Other’ patients having increased odds (1.36 and 1.29, respectively) of having a UDI than White patients, after adjusting for age. Unsurprisingly, the age-adjusted analyses also showed that LP and UDI were strongly associated (odds ratio (OR) 8.03, 95% confidence interval (CI) 7.38–8.74). Among late presenters, Asians had lower odds of UDI (OR 0.77, 95% CI 0.61–0.99) than Whites. For non-late presenters, the odds of UDI were higher in Asians (OR 1.18, 95% CI 1.09–1.28), Blacks (OR 1.47, 95% CI 1.32–1.64), and ‘Other’ (OR 1.30, 95% CI 1.08–1.57) compared to Whites (ethnicity and LP interaction *P* < 0.001). UDI was also associated with being more deprived, having an acute kidney injury, diabetes, higher comorbidity burden and history of cancer, heart failure, myocardial infarction, and minimal engagement with outpatient services before starting dialysis.

**Conclusions:**

Our study highlighted ethnicity as an important risk factor for UDI. Further, timing of presentation appears to alter ethnic differences in UDI.

## Graphical Abstract

**Graphical Abstract:**
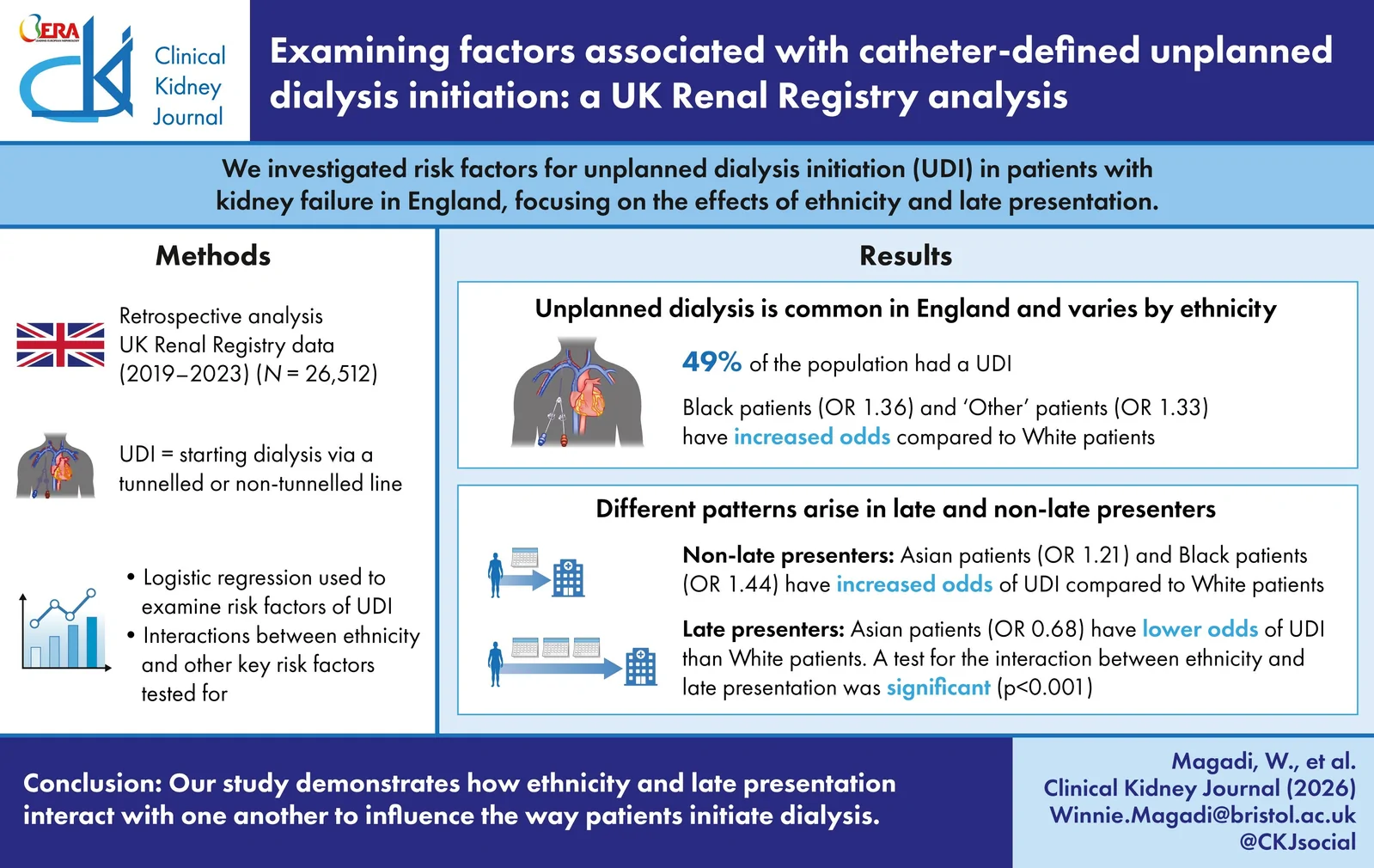

KEY LEARNING POINTS **What was known:**

- Presenting late to a nephrologist is known to strongly predict unplanned dialysis initiation (UDI), although this relationship is not so clear cut. There is also evidence of an association between ethnicity and late presentation (LP). However, limited studies in the UK have examined the association between ethnicity, LP, and UDI.

**This study adds:**

- Our study investigated the risk factors for UDI, focusing on the effects of ethnicity and late presentation. We found that being of Black and ‘Other’ ethnicity compared to White, increased one’s risk of UDI. Our study also shows that the effect of ethnicity on UDI varies by timing of presentation.

**Potential impact:**

- Our study highlights ethnicity as an important risk factor for UDI. Factors such as presenting late, higher deprivation, reduced contact with nephrology services, and presence of comorbidities, may all be on the causal pathway between ethnicity and UDI and should be investigated using the appropriate statistical methods.

## INTRODUCTION

Approximately half of patients with end-stage kidney disease (ESKD) in the UK who commence dialysis, do so in an unplanned or urgent manner [1]. Definitions of unplanned dialysis initiation (UDI) vary across studies and countries, but commonly include emergency or inpatient starts, use of central venous catheters (CVC) including tunnelled and non-tunnelled lines, and limited prior contact with renal services [2]. These inconsistent definitions make it difficult to compare studies, but UDI is consistently linked to worse outcomes including reduced quality of life, increased number of hospitalisations, and higher rates of mortality [3]. Late presentation to a nephrologist (LP) is often highlighted as a strong predictor of UDI [4], but many patients known to renal services and presenting early still experience UDI [5]. Other established risk factors include older age, lack of patient education, cardiovascular disease, and higher comorbidity burden [6]. More recently, studies have drawn attention to healthcare-related factors, such as reduced creatinine monitoring, incomplete chronic kidney disease (CKD) coding [2], and sociodemographic determinants such as living alone. Evidence on the effect of ethnicity on UDI remains limited and inconclusive [2]. Just two single-centre studies in the UK have explored this association, and demonstrated that South Asian or Black patients are less likely than White patients to experience an unplanned or urgent dialysis start [1, 7]. It is worth noting that UDI was defined as receiving nephrology care in the low clearance clinic for <90 days in one study, while the other distinguished between patients who required acute initiation of kidney replacement therapy (KRT) and were known to renal services for >90 days (unplanned starters) or <90 days (urgent starters).

Given the established association between LP and UDI, gaining a better understanding of how ethnicity and LP are related may provide insight into the barriers to a planned dialysis initiation. A recent report looking at disparities across sociodemographic groups among adults and children with kidney failure in the UK showed that rates of LP differ among ethnic groups [8]. Asians were the least likely to present late, consistent with less UDI, whilst those of Black or Other ethnic origin were the most likely to present late.

To our knowledge, no large-scale studies in the UK have examined how ethnicity impacts the likelihood of initiating dialysis in an unplanned manner. Thus, our study aimed to identify risk factors for UDI, i.e. catheter use when starting dialysis, in patients who initiated dialysis across England; specifically exploring the effects of ethnicity and LP on UDI, separately and jointly.

## MATERIALS AND METHODS

### Study cohort and design

We conducted a retrospective cohort analysis, utilising data from the UK Renal Registry (UKRR) on adults (age ≥18) initiating dialysis between 1 January 2019 and 31 December 2023.

The outcome was UDI, defined as initiating dialysis with a CVC—either a tunnelled or non-tunnelled line. A planned initiation referred to a patient who had definitive access at start: a surgically created arteriovenous fistula (AVF), arteriovenous graft (AVG), or a peritoneal dialysis (PD) catheter.

The main exposures of interest were ethnicity and late presentation. Ethnicity was based on self-reports by patients collected during routine clinical care and sent to UKRR. These data were then grouped in the categories used in the 2021 census for England and Wales: Asian, Black, Mixed, Other, and White. Presentation time was defined as the time between first seeing a nephrologist and starting KRT, based on the UKRR data. We classified non-late versus late presenters using a 90-day cut-off point.

### Registry and hospital data

The UKRR holds data on all ESKD patients who receive KRT from kidney centres in the UK. The study cohort was linked to Hospital Episode Statistics (HES), which contain a wide range of information on patient admissions and outpatient appointment attendances from all NHS hospitals in England. We used the HES linkage to derive information on comorbidities and calculate the Charlson Comorbidity Index (CCI) score [9], outpatient attendances and admissions related to nephrology, and diagnosis codes for acute kidney injury (AKI) (ICD10 code N17). Regarding comorbidities, we used the HES data from 3 years prior to dialysis initiation. For AKI, outpatient attendances, and inpatient admissions, we searched the records in the 2 years prior to dialysis start, excluding those that occurred during the period of dialysis initiation. We determined whether a patient attended their scheduled appointment based on the relevant field in HES (see appendix 1) and grouped them into (i) high attendance (10 or more appointments), (ii) low attendance (less than 10 appointments), (iii) did not attend (those that had an outpatient appointment scheduled, but did not attend any), (iv) no outpatient appointments, but may have had an inpatient admission related to nephrology in the 2 years prior to starting dialysis.

Data on vascular access were obtained from the UKRR. In cases where the data were missing, we derived access codes from HES (see appendix 2), to determine whether patients initiated with temporary or permanent access. We looked for access codes in both the inpatient and outpatient datasets and limited the search to 6 months prior to starting dialysis, using the last recorded access code to assign patients. In cases where a patient had both permanent and temporary access codes recorded on the same day, we kept the permanent access codes. We then tested for agreement between the UKRR and HES data using a quadratic weighted Kappa statistic [10]. Given the varying definitions adopted for UDI in the literature, we also compared access data with data on patients who started either as an inpatient or outpatient.

The deprivation data were based on the indices of multiple deprivation (IMD 2019), which we obtained by linking the Registry patient postcode with the ONS Postcode Directory. IMD is the official measure of area-level deprivation in England and is based on seven domains derived from the UK census: income, employment, education, health, crime, barriers to housing and services, and living environment. IMD have been categorised into five quintiles for our analysis (1 being the most deprived and 5 being the least) [11].

Data on other risk factors (age, sex, and primary kidney disease) were obtained from the UKRR dataset.

### Statistical analysis

We ran univariable and multivariable logistic regression analyses to establish the risk factors for UDI. First, we examined the effect of age, sex, ethnicity, deprivation, late presentation, cause of kidney disease, diabetes, cancer, myocardial infarction (MI), heart failure, number of comorbidities, AKI, and attendance of nephrology appointments on UDI separately. For the adjusted analyses, and based on the relationships between the various risk factors for UDI depicted in the directed acyclic graph in Fig. 1, we only accounted for age in the models. While age did not ‘cause’ ethnicity, the distribution of age differed amongst ethnic groups, with a younger population seen in ethnic minorities with kidney disease compared to Whites [8]. Further, many of the other factors we studied appeared to mediate the association between ethnicity and UDI (including late presentation), rather than confound the relationship. To account for variation in rates of UDI between centres, we used multilevel models with centre specified as a random intercept. We then carried out sensitivity analyses to determine whether limiting our analyses only to haemodialysis (HD) patients only impacted the results. This is worth investigating, given that PD patients were far less likely to initiate dialysis as unplanned, which is mainly due to presenting earlier.

**Figure 1:**
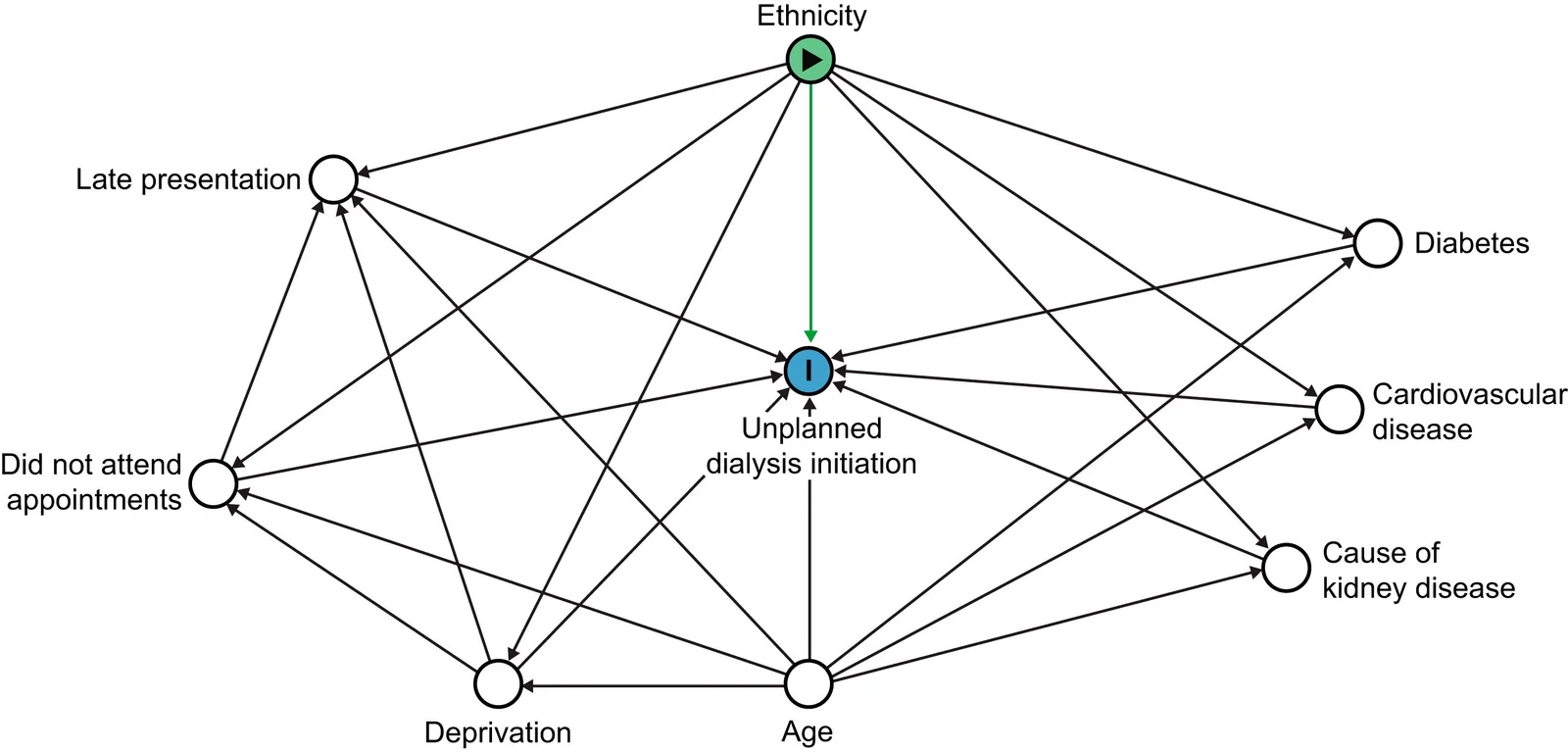
Directed acyclic graph to depict associations among risk factors for unplanned dialysis initiation.

To determine whether the effect of ethnicity on UDI differed amongst non-late and late presenters, we stratified the analysis by timing of presentation. We also tested for an interaction between ethnicity and each of LP, age and sex, using a likelihood ratio test.

Regarding outcomes, we conducted a chi-square test to assess whether ethnicity and LP were associated, based on the previous literature and given the strong association between LP and UDI [6]. We conducted all analyses in SAS 9.4 (SAS Institute, Cary, NC, USA).

## RESULTS

A total of 32 157 patients started dialysis in England between 1 January 2019 and 31 December 2023. We excluded those with missing data on vascular access (*N* = 2509), ethnicity (*N* = 202), presentation (*N* = 636), and those that were not linked to HES (*N* = 2298). This included patients who did not have any admission recorded in HES between 1997 and 2023 (*N* = 1098) or opted out/had incorrect IDs (*N* = 1200). Therefore, 26 512 patients were included in the final analyses. The flow of patients is shown in Fig. 2. We compared the characteristics of patients excluded because of missing vascular access data with those included in the final cohort (Table S1). The two groups were broadly similar; however, excluded patients were more likely to present late and have less contact with outpatient nephrology services.

**Figure 2:**
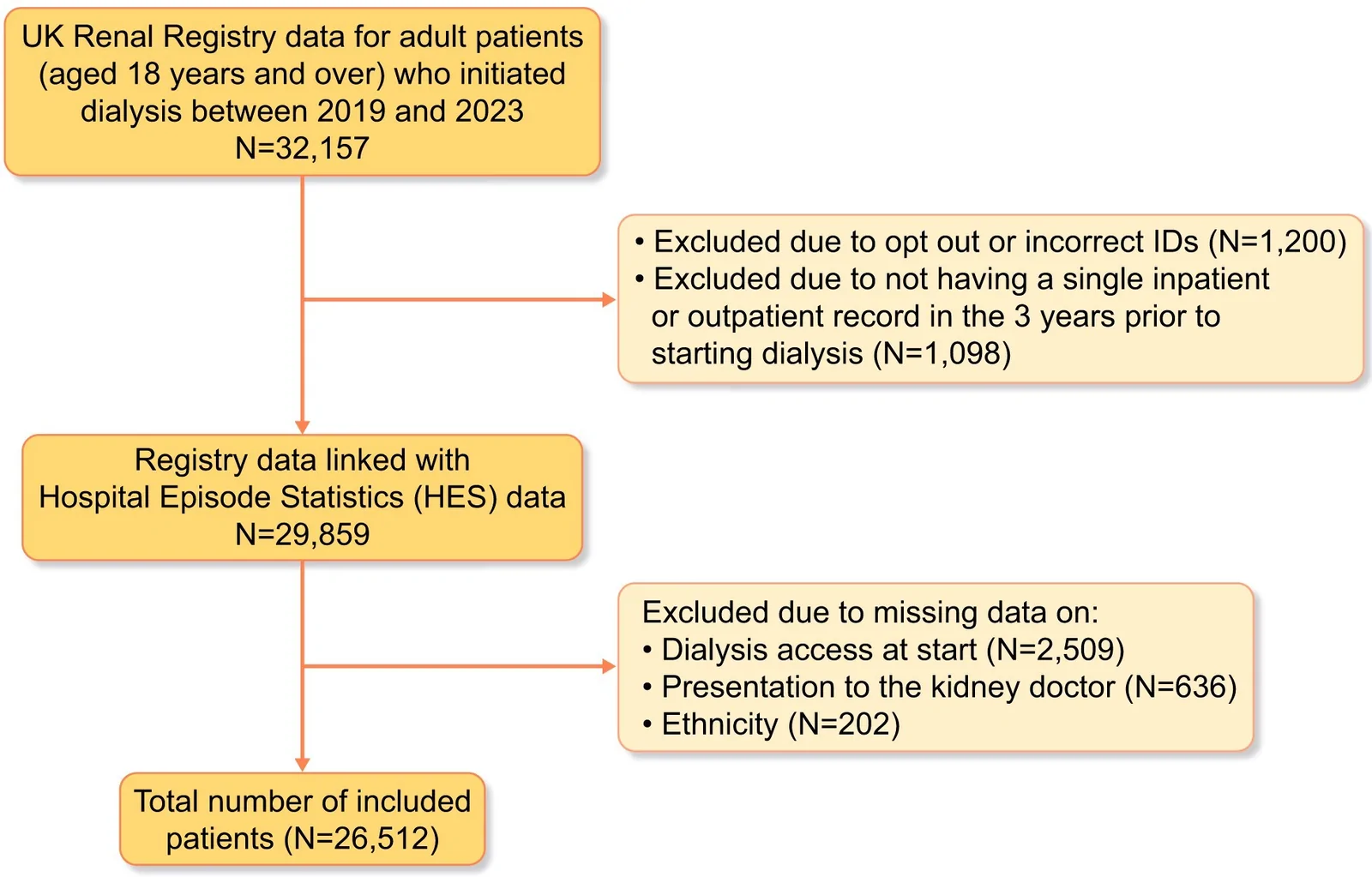
Flow of patient selection.

Regarding the use of HES data, agreement between the UKRR and HES vascular access data was strong [weighted kappa *K*_W_ = 0.76, 95% confidence interval (CI) (0.75, 0.76)]. These results are presented in Table S2.

### Patient and clinical characteristics

Just under half of the cohort were aged 65 years and over (*N* = 12 868, 49%), 65% were male (*N* = 17 164), 71% from a White background (*N* = 18 904), and about half were in the two most deprived quintiles (*N* = 13 047, 49%) (see Table 1). The most common primary cause of kidney disease was diabetes, representing 30% of the population (*N* = 7989), while 49% (*N* = 13 060) either had this condition listed as a comorbidity or primary cause. About half of the study population had at least one comorbidity aside from their CKD (*N* = 13 582), which included heart failure (*N* = 6379, 24%), MI (*N* = 3564, 13%), and cancer (*N* = 2723, 10%). The vast majority were non-late presenters (*N* = 21 764, 82%), and half of the patients experienced at least one AKI episode in the 2 years prior to initiating dialysis (*N* = 13 353, 50%). In the 2 years leading up to dialysis initiation, 32% had low outpatient attendance (*N* = 8442), 56% had high attendance (*N* = 14 909), 1% did not attend any scheduled appointments (*N* = 251, 1%), and 11% had no outpatient appointments prior to initiating dialysis (*N* = 2910, 11%).

**Table 1:** Demographics and clinical characteristics of patients who initiated dialysis between 2019 and 2023.

		*N (%)*	% of unplanned dialysis starters
	Total	26 512 (100)	49.4
Age group (years)	18–39	2899 (10.9)	52.6
	40–64	10 745 (40.5)	49.1
	65–74	6701 (25.3)	48.8
	≥75	6167 (23.3)	48.9
Sex	Male	17 164 (64.7)	49.5
	Female	9348 (35.3)	49.2
Ethnicity	Asian	4209 (15.9)	49.3
	Black	2309 (8.7)	56.0
	Mixed	460 (1.7)	50.7
	Other	630 (2.4)	55.4
	White	18 904 (71.3)	48.3
Deprivation quintile	1 (most deprived)	6961 (26.3)	53.1
	2	6086 (23)	50.6
	3	5253 (19.8)	49.2
	4	4456 (16.8)	46.0
	5 (least deprived)	3713 (14)	44.6
	Missing	43 (0.2)	60.5
Primary kidney disease	Diabetes	7989 (30.1)	48.9
	Glomerular disease	2862 (10.8)	41.5
	Hypertension	1886 (7.1)	46.5
	Polycystic kidney disease	1323 (5)	26.0
	Pyelonephritis	1177 (4.4)	48.9
	Renovascular disease	1076 (4.1)	46.4
	Other	4039 (15.2)	63.4
	Uncertain	3725 (14.1)	47.4
	Missing	2435 (9.2)	56.4
Diabetes	No/Missing	13 452 (50.7)	48.0
	Yes	13 060 (49.3)	50.8
Cancer	No	23 789 (89.7)	48.1
	Yes	2723 (10.3)	60.7
Myocardial infarction	No	22 948 (86.6)	48.2
	Yes	3564 (13.4)	56.6
Heart failure	No	20 133 (75.9)	45.5
	Yes	6379 (24.1)	61.4
No. of comorbidities	0	12 930 (48.8)	41.6
	1–2	8246 (31.1)	52.7
	3+	5336 (20.1)	63.1
Presentation	Non-late	21 764 (82.1)	41.7
	Late	4748 (17.9)	84.3
Acute kidney injury	No	13 159 (49.6)	34.8
	Yes	13 353 (50.4)	63.8
Contact with nephrology services	≥10 appointments	14 909 (56.2)	31.0
	<10 appointments	8442 (31.8)	65.1
	Admissions only	2910 (11)	94.0
	Did not attend appointment(s)	251 (1)	92.4

### Factors associated with unplanned dialysis initiation

Nearly half of the cohort started dialysis in an unplanned manner (*N* = 13 087, 49%) (see Table 1), with the proportion ranging from 19% to 62% between kidney centres (Fig. S1). A significant proportion of patients started dialysis during a hospitalisation (*N* = 11 967, 45.1%), rising to 70.7% amongst those who had a UDI.

For patients who presented to their nephrologist in a timely fashion (*N* = 21 764), 42% initiated dialysis as unplanned compared with 84% for late presenters (see Table 1). A large proportion of unplanned starters were also seen in the group with a higher comorbidity burden, i.e. three or more comorbidities (63%) versus those with none (42%). Regarding patients who had an AKI prior to initiating dialysis, 64% had a UDI compared with 35% for those that did not have an AKI. The level of contact with renal services prior to starting dialysis also appeared to strongly predict outcomes, given that UDI rates among those that had a low level of appointment attendance (65%) was more than double the rate that attended a high number of appointments (31%). These difference were increased further for those that did not attend any outpatient appointment (92%) or had no appointments recorded (94%).

### Ethnicity, late presentation, and unplanned dialysis initiation

Our unadjusted analyses revealed that ethnicity and UDI were associated, with patients of Black or Other origin being more likely to have an unplanned start than their White counterparts [odds ratio (OR) 1.36, 95% CI 1.24–1.50 and OR 1.29, 95% CI 1.1–1.52, respectively]. We also observed a strong effect of late presentation on UDI (OR 8.03, 95% CI 7.38–8.74). These associations were maintained after adjusting for age (see Table 2) and limiting the analyses to HD patients only (Table S3). However, while ethnicity and LP remained as strong predictors of unplanned HD initiation, Asians were significantly less likely to have a UDI than Whites. The latter result was non-significant when we combined HD and PD patients.

**Table 2:** Univariable and multivariable logistic regression analysis of the risk factors for unplanned dialysis initiation (2019–2023).

Variable	Categories	UnadjustedOR (95% CI)	Unadjusted*P*-value	Age adjusted (multilevel)OR (95% CI)	Age adjusted*P*-value
Age group	18–39 years (Ref)		0.004	N/A	N/A
	40–64 years	0.87 (0.8–0.94)			
	65–74 years	0.86 (0.79–0.94)			
	≥75 years	0.87 (0.79–0.95)			
Sex	Male (Ref)		0.692		0.758
	Female	0.99 (0.94–1.04)		0.99 (0.94–1.04)	
Ethnicity	White (Ref)		<0.0001		<0.0001
	Asian	1.04 (0.97–1.11)		0.99 (0.92–1.07)	
	Black	1.36 (1.24–1.48)		1.36 (1.24–1.5)	
	Mixed	1.1 (0.91–1.32)		1.2 (0.99–1.46)	
	Other	1.33 (1.13–1.56)		1.29 (1.1–1.52)	
Deprivation quintile (IMD)	1—Most deprived (Ref)		<0.0001		<0.0001
	2	0.9 (0.84–0.97)		0.87 (0.81–0.94)	
	3	0.86 (0.8–0.92)		0.83 (0.77–0.89)	
	4	0.75 (0.7–0.81)		0.74 (0.69–0.8)	
	5—Least deprived	0.71 (0.66–0.77)		0.69 (0.64–0.76)	
Primary kidney disease	Polycystic kidney disease (Ref)		<0.0001		<0.0001
	Diabetes	2.73 (2.39–3.11)		2.8 (2.45–3.2)	
	Glomerular disease	2.02 (1.75–2.33)		2 (1.73–2.32)	
	Hypertension	2.47 (2.12–2.88)		2.55 (2.18–2.98)	
	Pyelonephritis	2.72 (2.3–3.22)		2.86 (2.41–3.39)	
	Renovascular disease	2.46 (2.07–2.92)		2.75 (2.31–3.28)	
	Other	4.92 (4.28–5.65)		5.21 (4.52–6)	
	Uncertain	2.56 (2.23–2.94)		2.68 (2.33–3.09)	
	Missing	3.69 (3.18–4.27)		4.78 (4.08–5.59)	
Diabetes	Diabetes^a^—No/Miss (Ref)		<0.0001		<0.0001
	Yes	1.12 (1.07–1.18)		1.14 (1.09–1.2)	
Cancer	Cancer—No (Ref)		<0.0001		<0.0001
	Yes	1.67 (1.54–1.81)		1.79 (1.65–1.95)	
Heart failure	Heart failure—No (Ref)		<0.0001		<0.0001
	Yes	1.9 (1.8–2.02)		2.07 (1.95–2.2)	
Myocardial infarction	Myocardial infarction—No (Ref)		<0.0001		<0.0001
	Yes	1.4 (1.3–1.5)		1.48 (1.38–1.6)	
Comorbidities	No comorbidities (Ref)		<0.0001		<0.0001
	1–2	1.57 (1.49–1.66)		1.76 (1.66–1.87)	
	3+	2.4 (2.25–2.57)		2.87 (2.67–3.08)	
Presentation	Non-late (Ref)		<0.0001		<0.0001
	Late	7.51 (6.92–8.16)		8.03 (7.38–8.74)	
Acute kidney injury	AKI—No (Ref)		<0.0001		<0.0001
	Yes	3.3 (3.14–3.47)		3.42 (3.24–3.6)	
Contact with nephrology services	≥10 appointments^b^ (Ref)		<0.0001		<0.0001
	<10 appointments	4.16 (3.93–4.4)		5.37 (5.04–5.72)	
	Admissions only	34.78 (29.73–40.68)		44.56 (37.95–52.33)	
	Did not attend appointment(s)	27.18 (17.01–43.45)		31.96 (19.92–51.29)	

^a^ Diabetes recorded either as a primary cause of kidney disease or comorbidity.

^b^ Nephrology outpatient attendance in the 2 years preceding dialysis start.

The stratified analyses showed that the effect of ethnicity on UDI differed by timing of presentation, after accounting for age (see Fig. 3). Amongst non-late presenters, Asian (OR 1.18, 95% CI 1.09–1.28), Black (OR 1.47, 95% CI 1.32–1.64), and ‘Other’ (OR 1.30, 95% CI 1.08–1.57) patients were more likely to start as unplanned than Whites. For late presenters, Asians were less likely than Whites to have a UDI (OR 0.77, 95% CI 0.61–0.99). These findings suggest that late presentation amplifies UDI risk in White patients more than in Asian patients (see Fig. 4). Thus, we compared demographics and clinical characteristics of these two groups in both late and non-late presenters (Table S4). Evidently, Asians who present late are younger and less deprived than those who present in a timely manner. These patterns were not observed for Whites; however, we did see that late presenters had a higher comorbidity burden among this group.

**Figure 3:**
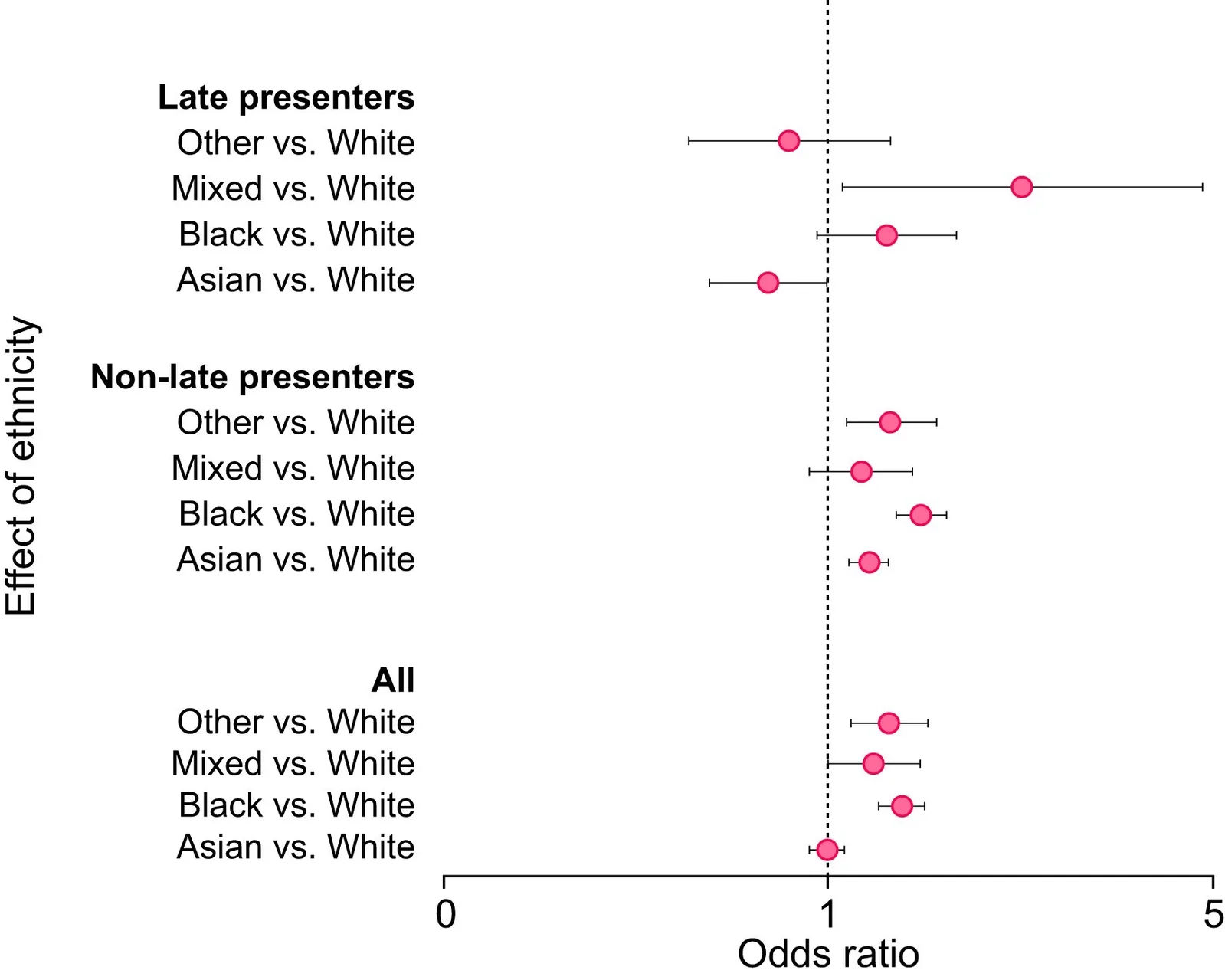
Multivariable logistic regression the effect of ethnicity on unplanned dialysis, by timing of presentation.

**Figure 4:**
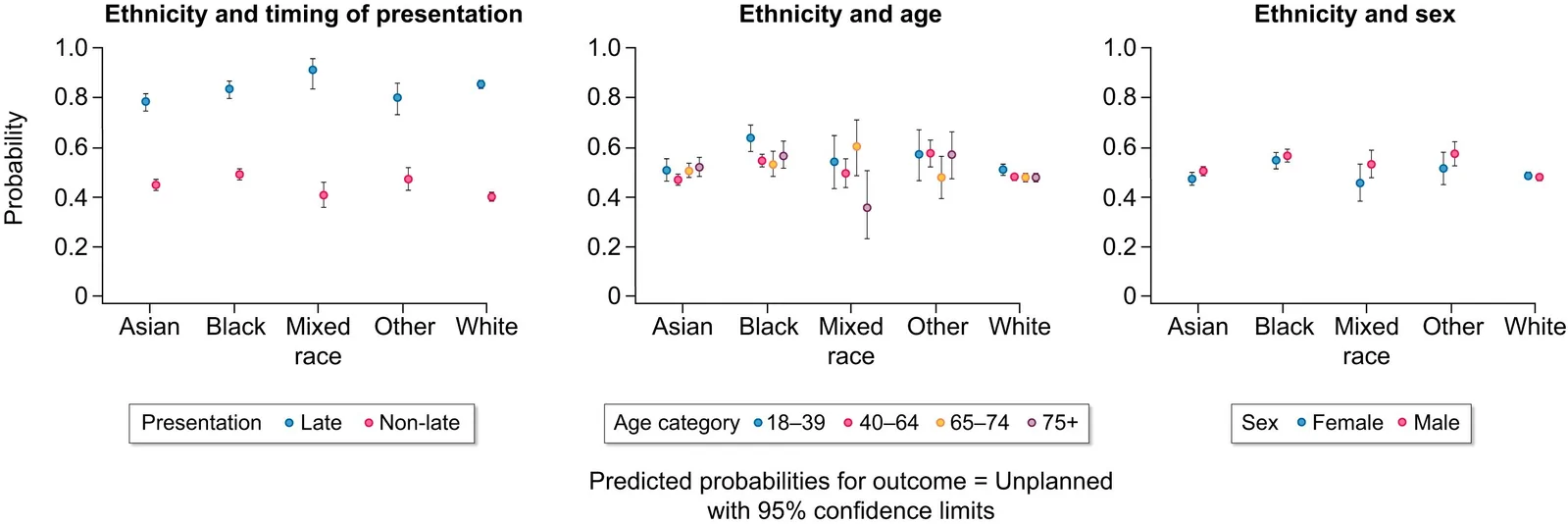
Tests of interactions between ethnicity and each of age, sex, and timing of presentation.

A test for the interaction between ethnicity and LP showed that timing of presentation moderates the effect of ethnicity on UDI for Asians, Blacks, and ‘Other’ (*P* < 0.001). Detailed results are presented in Fig. 4. We also observed interactions between ethnicity and age (*x*² (4) = 73.1, *P* < 0.001), and ethnicity and sex (*x*² (4) = 68.4, *P* < 0.001). Specifically, age moderates the effect of ethnicity on UDI amongst Blacks, while sex moderates this effect for Asians.

Regarding the analysis of the association between ethnicity and timing of presentation, we found that the two factors were strongly associated [*x*² = 87.6(4), *P* < 0.001] with Asians being the least likely to present late (13%) and those classed as 'Other' being the most likely (25%).

### Other risk factors for unplanned dialysis initiation

The unadjusted and adjusted analyses showed associations between UDI and being younger, more deprived, having any cause of kidney disease compared with polycystic kidney disease, a history of cancer, congestive heart failure and MI, higher comorbidity burden, having AKI, and low/no attendance of nephrology-related outpatient appointments, leading up to dialysis initiation (see Table 2).

## DISCUSSION

This is the first large-scale study to examine the risk factors for UDI across all kidney centres in England. Given the poor outcomes experienced by patients who start dialysis in an unplanned manner, it is crucial to understand the scale of the problem and factors associated with UDI. We found that nearly half of the patients who initiated dialysis were unplanned, consistent with previous literature. This is despite many patients presenting early to the nephrologist, highlighting the complexities of tackling the issue of UDI. It is evident that a large proportion of patients with ESKD have other serious underlying health conditions that may lead to a sudden deterioration of their kidney function or other acute complications [12]. Therefore, it is inevitable that some of the sicker patients start dialysis as an emergency, even in cases where they are seen regularly by health professionals.

The risk factors for UDI identified in our study were mostly in line with previous research, including late presentation, presence of cardiovascular disease, comorbid illness, lower socioeconomic status, and limited contact with nephrology services prior to initiating dialysis [1, 2]. Our findings also highlighted ethnicity as a predictor for UDI, with Black and ‘Other’ patients more likely than White patients to initiate unplanned dialysis. Studies in the US using national registry data have shown similar trends. Copeland *et al*. demonstrated that Black patients were not only less likely to have an AVF than White patients, they experienced higher rates of access failure, and at an earlier stage than White patients [13]. This is despite adjusting for the type of access used at start of dialysis, age, and comorbidity burden. One of the explanations is that Black individuals are known to receive less pre-dialysis care, due to the inequity in access to health care in the US. However, the evidence in the UK regarding the effect of ethnicity on UDI is mixed and limited. In a single centre study based in East London, Olaitan *et al*. found that Blacks and Asians were less likely than Whites to have an unplanned dialysis start, but these differences disappeared after adjusting for other known risk factors of UDI, e.g. age, comorbidity burden, and several factors related to nephrology care [7].

Another key finding in our study was the varying effect of ethnicity on UDI, by timing of presentation. Specifically, we demonstrated that among non-late presenters, Asian, Black, and ‘Other’ individuals were more likely to have a UDI than Whites. However, a high risk of UDI was observed among late-presenting White patients, given that UDI was less likely to occur in Asians than Whites who presented late to the nephrologist. Thus, late presentation appears to carry unequal consequences across ethnic groups, which may partly be explained by differences in age, deprivation, and comorbidity amongst late and non-late presenters. We found that late-presenting Asians were younger, less deprived, and had fewer comorbidities compared to late-presenting Whites. For patients known to renal services, the literature suggests that despite being more likely to present early, poorer health outcomes (including starting dialysis with temporary vascular access) are often experienced by Asians compared to Whites [14, 15]. The reasons for this are not clear, but there is evidence to suggest that faster progression of CKD, higher comorbidity burden, i.e. diabetes, and social determinants of health are contributing factors [16]. Further, it is possible that lower levels of health literacy and mistrust of the healthcare system may influence health-related decision-making and are often important considerations among ethnic minority groups [18–20].

In relation to the association between ethnicity and LP, previous studies report inconsistent findings as some suggest a higher risk among ethnic minority groups [21, 22], while indicating that the risk is greater in White patients [23]. Differences in adjustments for age, comorbidity burden, deprivation, and other known risk factors may partly explain these discrepancies. In our study and in another recent UK Renal Registry analysis, late presentation was found to be least common in Asians, and most common in those who were classed as ‘Other’ [24].

Regarding the effect of age on UDI, we observed that younger patients were more likely to initiate dialysis in an unplanned manner, consistent with two recent large-scale studies using the French Registry data [25, 26]. It has been hypothesised that because younger individuals have a lower comorbidity burden, they are less likely to visit their GP, which eventually leads to delayed referral and unplanned starts [26]. However, in a review of the risk factors of UDI by Hassan *et al*., only one study reported younger age as an independent risk factor [6]. This study was limited to home haemodialysis patients only, making it difficult to generalise the results to the entire dialysis population. Many of the included studies in the review showed an association between older age and UDI, which is partly explained by how nephrologists approach decisions about care for the elderly due to frailty [6, 27].

Given the findings of our study, more research is needed to better understand how the risk factors for UDI interact with one another. Causal mediation analysis (CMA) is a technique used to examine the causal mechanisms underlying the association between an exposure and outcome, and for identifying whether specific mediators explain some of this relationship [28]. Few studies have adopted this approach to assess relationships among the known risk factors of UDI in patients with advanced CKD. Using the data of the Renal Epidemiology and Information Network (REIN) registry in France, one study using CMA found that the effect of sex on UDI was mediated by pre-dialysis care, particularly for deprived patients [29].

### Strengths and limitations

The definition we used for UDI was based on dialysis access at initiation. This is a good indicator of how prepared a patient is when starting dialysis, but does not reflect that starting with a CVC is not a preferred option for all patients. Our definition may introduce misclassification bias, given that some patients intentionally initiate HD using a catheter despite prolonged nephrology follow-up, particularly elderly or frail patients. AVF creation often yields poor outcomes in older patients, including higher rates of non-maturation, longer times to functional use, and interventions that are more complex [30]. Thus, the inferences from our study are limited to vascular access at initiation, and the relationship between ethnicity, late presentation, and UDI may differ for alternative definitions.

Our study was strengthened by the use of UKRR data which cover the entire chronic KRT population in England. This provided a sufficiently large cohort to detect ethnic differences and identify disparities in UDI. However, there were a few limitations to the data available. The use of routinely collected clinical data is known to have some inaccuracies and omissions [31]. The UKRR also lacks information on some of the important risk factors for UDI, e.g. health literacy and pre-dialysis care. Further, missing data on vascular access may have resulted in selection bias, although this was partially mitigated through linkage with HES, which improved completeness of vascular access codes and provided additional information on comorbidities.

## CONCLUSION

Our study was the first large-scale study in England to demonstrate how ethnicity and late presentation interact with one another to influence the way patients initiate dialysis. The finding that UDI risk is increased in ethnic minority groups among early presenters, but the reverse is observed in late presenters, is one that requires further investigation. Given the limitations of UKRR data, more prospective cohort studies are needed to investigate novel determinants of UDI, as well as the role of mediating factors in explaining the association between ethnicity and UDI. Further research should also delve deeper into ethnic-specific barriers, as well as early engagement, to help tackle the high rates of UDI observed in the population.

## ETHICS APPROVAL

The UKRR holds permissions under s251 of the National Health Service (NHS) Act 2006, to gather, process, and share confidential patient information for the purposes of audit and research. These permissions are renewed annually by the UK’s Health Research Authority’s Confidentiality Advisory Group. The collection and analysis of the data for research were carried out under the ethics permissions granted to the UKRR by the Research Ethics Committee (Ref: 16/NE/0042). The authors excluded patients who opted out of their confidential information being used for research.

## Supplementary Material

sfag287_Supplemental_File

## Data Availability

The data underlying this article will be shared on reasonable request to the corresponding author. The data underlying this study were obtained from the UK Renal Registry (UKRR) and linked to Hospital Episode Statistics (HES). Access to these data is restricted. Researchers interested in UKRR data should apply through the UK Renal Registry’s data application process. Further details about the application process and access conditions can be found at [https://www.ukkidney.org/audit-research/how-access-data/ukrr-data]. Researchers interested in accessing these data should submit a formal application, which will be reviewed following the UK Renal Registry’s governance and ethical approval policies.

## Appendix 1 List of codes in HES to indicate whether or not a patient attended their appointment.

**Table:**

Code number	Description
1	Attended first appointment
2	Attended subsequent appointment
3	Attended but first/subsequent/tele unknown
4	Did not attend first appointment
5	Did not attend subsequent appointment
6	Did not attend but first/subsequent/tele unknown
7	Patient cancelled first appointment
8	Patient cancelled subsequent appointment
9	Patient cancelled but first/subsequent/tele unknown
10	Hospital postponed/cancelled first appointment
11	Hospital postponed/cancelled subsequent appointment
12	Hospital postponed/cancelled but first/subsequent unknown
13	Not known
21	Attended first tele consultation (from 2008–09)
22	Attended subsequent tele consultation (from 2008–09)
24	Did not attend first tele consultation (from 2008–09)
25	Did not attend subsequent tele consultation (from 2008–09)
27	Patient cancelled first tele consultation (from 2008–09)
28	Patient cancelled subsequent tele consultation (from 2008–09)
30	Hospital postponed/cancelled first tele consultation (from 2008–09)
31	Hospital postponed/cancelled subsequent tele consultation (from 2008–09)

## Appendix 2 Dialysis access diagnostic and procedure codes recorded in Hospital Episode Statistics (HES).

**Table:**

System	Code	Name
OPCS-4	L74.1	Insertion of arteriovenous prosthesis
	L74.2	Creation of arteriovenous fistula
	L74.3	Attention to arteriovenous shunt
	L74.4	Banding of arteriovenous fistula
	L74.5	Thrombectomy of arteriovenous fistula
	L74.6	Creation of graft fistula for dialysis
	L74.8	Other specified arteriovenous shunt
	L74.9	Unspecified arteriovenous shunt
	X41.1	Insertion of ambulatory peritoneal dialysis catheter
	X41.2	Removal of ambulatory peritoneal dialysis catheter
	X42.1	Insertion of temporary peritoneal dialysis catheter
	L91.5	Can’t find
	X41.8	Other specified placement of ambulatory apparatus for compensation for renal failure
	X41.9	Unspecified placement of ambulatory apparatus for compensation for renal failure
	X42.8	Other specified placement of other apparatus for compensation for renal failure
	X42.9	Unspecified placement of other apparatus for compensation for renal failure
ICD10	Z49.0	Preparatory care for renal dialysis
	Z49.01	Encounter for fitting and adjustment of extracorporeal dialysis catheter
	Z49.02	Encounter for fitting and adjustment of peritoneal dialysis catheter
	T82.4	Mechanical complication of vascular dialysis catheter
	T82.41	Breakdown (mechanical) of vascular dialysis catheter
	T82.42	Displacement of vascular dialysis catheter
	T82.43	Leakage of vascular dialysis catheter
	T82.49	Other complication of vascular dialysis catheter
